# Medication-related osteonecrosis of the jaws: a series of 22 cases highlighting their histopathological features

**DOI:** 10.1590/1807-3107bor-2025.vol39.058

**Published:** 2025-06-02

**Authors:** Dandara Andrade de SANTANA, Yann Victor Paiva BASTOS, Paulo Tambasco de OLIVEIRA, Bruno Cunha PIRES, Flávia Caló Aquino XAVIER, Águida Cristina Gomes Henriques LEITÃO, Larissa Abbehusen COUTO, André Sampaio SOUZA, Luciana Maria Pedreira RAMALHO, Patrícia Ramos CURY, Jean Nunes dos SANTOS

**Affiliations:** (a) Universidade Federal da Bahia – UFBA, School of Dentistry, Dentistry and Health Postgraduate Program, Salvador, BA, Brazil.; (b) Private Orthodontics Office, Maceió, AL, Brazil.; (c) Universidade de São Paulo – USP, School of Dentistry of Ribeirão Preto, Cell Culture Laboratory, University of São Paulo, Ribeirão Preto, SP, Brazil.; (d) Centro Diagnóstico Pires, Feira de Santana, BA, Brazil.; (e) Universidade Federal da Bahia – UFBA, School of Dentistry, Laboratory of Oral and Maxillofacial Pathology, Salvador, BA, Brazil.; (f) Universidade Federal de Pernambuco – UFPE, School of Dentistry, Department of Clinic and Preventive Dentistry, Recife, PE, Brazil.; (g) Hospital Manoel Victorino, Salvador, BA, Brazil.; (h) Oncology Center of Bahia, Salvador, BA, Brazil.; (i) Department of Periodontology, Federal University of Bahia, Salvador, Bahia, Brazil.

**Keywords:** Osteonecrosis, Actinomycosis, Infections, Jaw

## Abstract

This study aimed to assess the clinicopathological characteristics of medication-related osteonecrosis of the jaw (MRONJ), addressing histopathological features and bone structure. Twenty-two MRONJ patients were retrospectively evaluated for osteonecrosis, osteomyelitis, bacterial colonization, bone resorption, reactive bone, osteon-like structure, lamellar bone, and basophilic lines. Specific staining and fluorescence and polarized light microscopy assessments were also performed. The mandible was significantly more affected by MRONJ. There was a predominance of African-Brazilian women aged sixty and older. Osteomyelitis was present in 82% of patients. Actinomycosis was observed in 36.4% of MRONJ patients. Osteoclasts were absent in MRONJ but bone resorption was often seen. Basophilic lines were observed in more than 80% of the patients. The MRONJ patients exhibited lamellar bone with osteon-like structures. This study provided important insight into MRONJ, demonstrating a diverse histopathological spectrum and infection with *Actinomyces* and fungi. The condition was characterized by a necrotized lamellar bone structure and more frequently affected the mandible of older women.

## Introduction

Osteonecrosis of the jaw has received much attention in recent decades because of the increasing number of cases related to the use of bisphosphonates, denosumab, and antiangiogenic drugs.^1,2^ Medication-related osteonecrosis of the jaw (MRONJ) is a process characterized by the exposure of necrotic bone for more than 8 weeks.^3,4^ Its frequency ranges from 0% to 6.9%.^5^

Bisphosphonates reduce the risk of fractures by inhibiting bone resorption.^6^ The mechanism of action of nitrogen-containing bisphosphonates is based on the inhibition of the mevalonate pathway, which produces farnesyl diphosphate and geranyl pyrophosphate. These substrates are required for the transcriptional modification of GTPases, enzymes that regulate osteoclast apoptosis.^7,8^ Denosumab is a human monoclonal antibody that binds with high affinity and specificity to RANKL, preventing the ligand from activating its single receptor, RANK, on the surface of osteoclasts and their precursors. Denosumab inhibits osteoclasts’ formation, activity, and survival, consequently reducing bone resorption.^9,10^ On the other hand, antiangiogenic agents are indicated for cancer treatment. These drugs comprise monoclonal antibodies or inhibitors of tyrosine kinase that directly or indirectly block the action of VEGF.^11,12^

Radiographically, MRONJ appears as osteosclerotic to completely osteolytic areas or even as mixed areas.^13,14^ Histologically, MRONJ is characterized by a reduction in vascular canals, empty bone marrow spaces, and areas of bone resorption where osteoclasts are present and osteocytes are absent.^12,15^

To our knowledge, few studies have focused on the clinicopathological characteristics of MRONJ.^16^ Despite its medication-related etiology, osteonecrosis may be a common feature of other conditions that affect the jawbones. However, MRONJ has clinical implications such as pain and can reduce the quality of life, particularly in patients with cancer.^24^ Therefore, this study aimed to assess the clinicopathological characteristics of MRONJ cases, highlighting the histopathological features and bone structure by polarized light and fluorescence microscopy.

## Methods

### Population, sample and patient selection

After approval by the Ethics Committee (Approval number 3.082.248), 36 patients diagnosed as MRONJ were retrieved from the archives of the Oral and Maxillofacial Surgical Pathology Service of the School of Dentistry, Federal University of Bahia, Salvador, Bahia, Brazil, and from the Head and Neck Clinical Center, Guatemala City, Guatemala. This was a retrospective, cross-sectional cohort study that was conducted in accordance with the Declaration of Helsinki. Data were collected between 2002 and 2019.

The inclusion criterion was having a description of medications and/or previous reports of cancer treatment. All data were obtained from clinical charts. After revision of clinical and histopathological features, 14 patients with MRONJ were excluded (five because of a history of radiotherapy, two because of the lack of sufficient biological material, and seven because of the lack of clinical data compatible with the histopathological diagnosis). All hematoxylin/eosin-stained (Merck, Darmstadt, DE) histological slides were evaluated by two examiners under a light microscope. Clinical data such as patient age, gender, location, color, symptoms, presence of infection, and swelling were obtained from the biopsy request forms. Radiographic data were also collected, if available. Finally, 22 patients with MRONJ were included in the study.

### Morphological analysis

Morphological features such as osteonecrosis, osteomyelitis, bacterial colonization (*Actinomyces* colonies), bone resorption, reactive bone, osteon-like structures, lamellar bone, and basophilic lines were evaluated in the slides of each case following the criteria adopted by previous authors.^25^ The morphological analysis was performed by an experienced pathologist (JNS).

### Specific staining

The specific histochemical stains Grocott-Gomori (Abcam, Cambridge, UK) were used to evaluate the presence of pathogens such as bacteria and fungi.

### Fluorescence and polarized light microscopy analyses

The slides were analyzed under a microscope (Leica DM 4000 B, Wetzlar, DE) coupled to a camera (Leica DFC310 FX, Wetzlar, DE) at 400x magnification. Eosin fluorescence was used for the analysis of the histological sections at maximal absorption and emission wavelengths of 527 and 550 nm, respectively.

### Data analysis

The collected data were entered into a Microsoft Excel^®^ spreadsheet. Descriptive analysis was performed by calculating the absolute and relative frequency of categorical variables and the mean and standard deviation (SD) of continuous variables.

## Results

### Clinical features

Among the 22 MRONJ patients, 18 were women (81.8%) and four were men (18.2%), with a female/male ratio of 4.5:1 (Table 1). Most patients were Afro-Brazilians and age ranged from 25 to 88 years [mean 68.3 years (SD ±15.84)]. There was a higher frequency of cases in the eighth decade of life.

**Table 1:** Demographic data of the 22 patients with medication-related osteonecrosis of the jaw.

Variabe	MRONJ*
	Mean ± SD, n (%)
Mean age	68.32 ±15.84
Sex	
Male	4 (18.2)
Female	18 (81.8)
Ancestry	
European	4 (25.0)
African-Brazilian	12 (75.0)
Location	
Mandible	12 (46.1)
Maxilla	9 (34.6)
Symptoms	
Painless	1 (38.5)
Painful	6 (61.5)
Infection	
Present	7 (87.5)
Absent	1 (12.5)
Swelling	
Present	2 (33.3)
Absent	4 (66.7)

*Medication-related osteonecrosis of the jaw. There are missing cases in all variables.

The mandible was significantly more affected than other sites (Table 1). Pain and swelling were found in 61.5% and 33.3% of patients, respectively (Table 1). The presence of infection was observed in 87.5% patients.

Nineteen of the 22 MRONJ patients were using nitrogen-containing bisphosphonates, two were using denosumab, and one was using antiangiogenic agents. The name of the associated drug was reported in only 10 patients. Zoledronic acid was the most frequently reported drug (n = 5), followed by risedronic acid (n = 3) and disodium ibandronate (n = 2).

### Radiographic features

**Figure 1:**
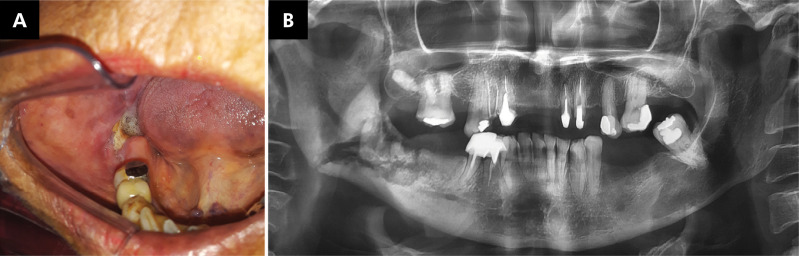
Medication-related osteonecrosis: A) Presence of exposed bone in the right alveolar ridge of the mandible. B) Panoramic radiograph showing alterations in bone density in the right posterior mandible. Note the bone sequestrum and vertical pathological fracture.

Radiographically, MRONJ patients had sclerotic areas around mandibular and maxillary teeth, including the toothless ridge, multiple radiolucencies, and local or generalized enlarged radiopacities with well-defined borders. Radiolucent images suggestive of osteonecrosis with well-defined margins were noted in some less extensive cases, while the margins were poorly defined in more extensive cases. Images suggestive of a bone sequestrum were observed in the center of the radiolucencies (Figure 1). The cortical bone was preserved. These radiographic findings were found in only nine MRONJ patients.

### Microscopic findings

MRONJ mainly appeared as irregular, often lamellar bone trabeculae or masses displaying osteon-like structures with no stromal tissue. “Thick and irregular” or “non-thick” basophilic lines were observed. These lines were parallel or non-parallel and contained empty bone lacunae, which often became larger in the absence of osteocytes.

The bone trabeculae observed in the MRONJ patients exhibited necrotic characteristics, including the presence of empty lacunae and empty medullary canals. The latter were colonized with bacteria and filled with inflammatory cells. Cup-shaped superficial bone resorption was present in 100% of the patients and involved more than 50% of the trabeculae in most patients. However, we did not observe osteoclasts in any of the cases.

Osteomyelitis was present in almost 82% of the MRONJ patients and was mainly acute (61.21%) and of mild intensity (45.45%) (Table 2).

**Table 2:** Histopathological features of the 22 patients with medication-related osteonecrosis of the jaw.

Variable	MRONJ* n(%)
Bone resorption	
Present	22 (100)
> 50%	17 (77.27)
< 50%	5 (22.72)
Osteomyelitis	
Present	14 (66.3)
Absent	8 (36.3)
Osteomyelitis intensity	
Intensity	2 (9.09)
Moderate	6 (27.27)
Mild	10 (45.45)
Absent	4 (18.18)
Osteomyelitis type	
Acute	11 (61.11)
Chronic	5 (27.77)
Mixed	2 (11.11)
Bacterial colonization	
Intensity	
Intense	17 (77.27)
Moderate	3 (13.63)
Mild	0
Absent	2 (9.09%)
*Actinomyces*	
Present	8 (36.36)
Absent	14 (63.63)
Basophilic lines	
Thick and irregular	18 (81.81%)
Non-thick	4 (18.18%)
Thick and irregular basophilic lines	
> 50%	7 (38.88%)
< 50%	11 (61.11%)
Reactive bone	
Present	1 (4.54%)
Absent	21 (94.45%)
Osteon-like structures	
Present	15 (%)
Absent	1 (4.45%)
Lamellar bone	
Present	21 (94.45%)
Absent	1 (4.45%)

*Medication-related osteonecrosis of the jaw.

Variable numbers of bacterial colonies were frequently observed amidst the bone trabeculae in MRONJ. These colonies were found around the necrotic bone, trapped inside the bone, or occupied the existing irregular medullary canals, which together with the inflammatory infiltrate culminated in bone sequestration. Bacterial colonization was present in 90.9% of the MRONJ patients and was classified as intense in most of them (77.27%). Actinomycosis was detected as the simultaneous occurrence of single and/or multiple colonies in the same specimen (Table 2).

A marked feature of these lesions was the presence of basophilic lines. They were “thick and irregular” in 81.8% of MRONJ patients (Table 2). Reactive bone was observed in only one patient (4.5%), while lamellar bone was present in almost 95% of patients. However, osteon-like structures were detected in only 9.1% of the MRONJ patients (Table 2).

**Figure 2:**
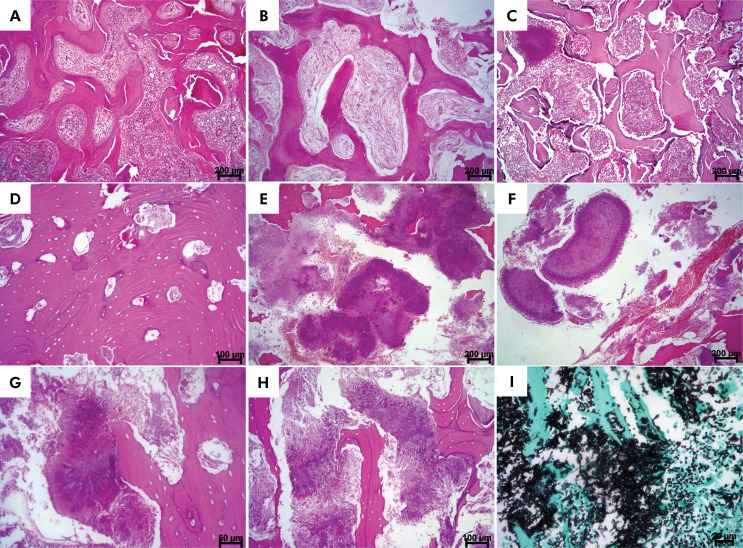
Medication-related osteonecrosis [hematoxylin and eosin, H&E]: A) Interconnected bone trabeculae that are curved and minimally cellular and interspersed with fibrous stroma. There are also basophilic lines arranged parallel to each other or non-parallel. Obliterated blood vessels surrounded by marked chronic inflammation (acute osteomyelitis) [scale bar: 200 µm] can be seen. B) Different area of the previous case showing curved lamellar bone trabeculae in stromal tissue with many empty lacunae, peritrabecular clefting, and no rimming of osteoblasts similar to fibrous dysplasia [scale bar: 200 µm]. C) Curved and interconnected bone trabeculae of variable thickness displaying peritrabecular clefting. Osteomyelitis, with fibrous stroma replaced by marked chronic inflammation can be seen. There are bacterial colonies compatible with *Actinomyces* in the upper left corner [scale bar: 200 µm]. D) Necrotic lamellar bone with empty lacunae of variable diameters. Note the osteon-like structures [scale bar: 100 µm]. I) Resorption [scale bar: 100 µm]. E) *Actinomyces* colonies exhibiting varied sizes and necrotic bone in the upper corner [scale bar: 200 µm]. F) Bacterial colonies (actinomycosis) with radiating clubs [scale bar: 200 µm]. G) Extensive accumulation of confluent bacterial colonies (actinomycosis). Necrotic bone with large bone lacunae [scale bar: 200 µm] is shown. H) Irregular necrotic bone trabeculae exhibiting thick basophilic lines parallel to each other and interspersed with irregular bacterial colonies. The bone surface with resorption is shown [scale bar: 200 µm]. I) Irregular necrotic bone amidst *fungi* infection compatible with *Aspergillosis* [scale bar: 50 µm; Grocott-Gomori] .

All histopathological features of MRONJ are shown in Figure 2.

### Fluorescence and polarized light microscopy analysis

**Figure 3:**
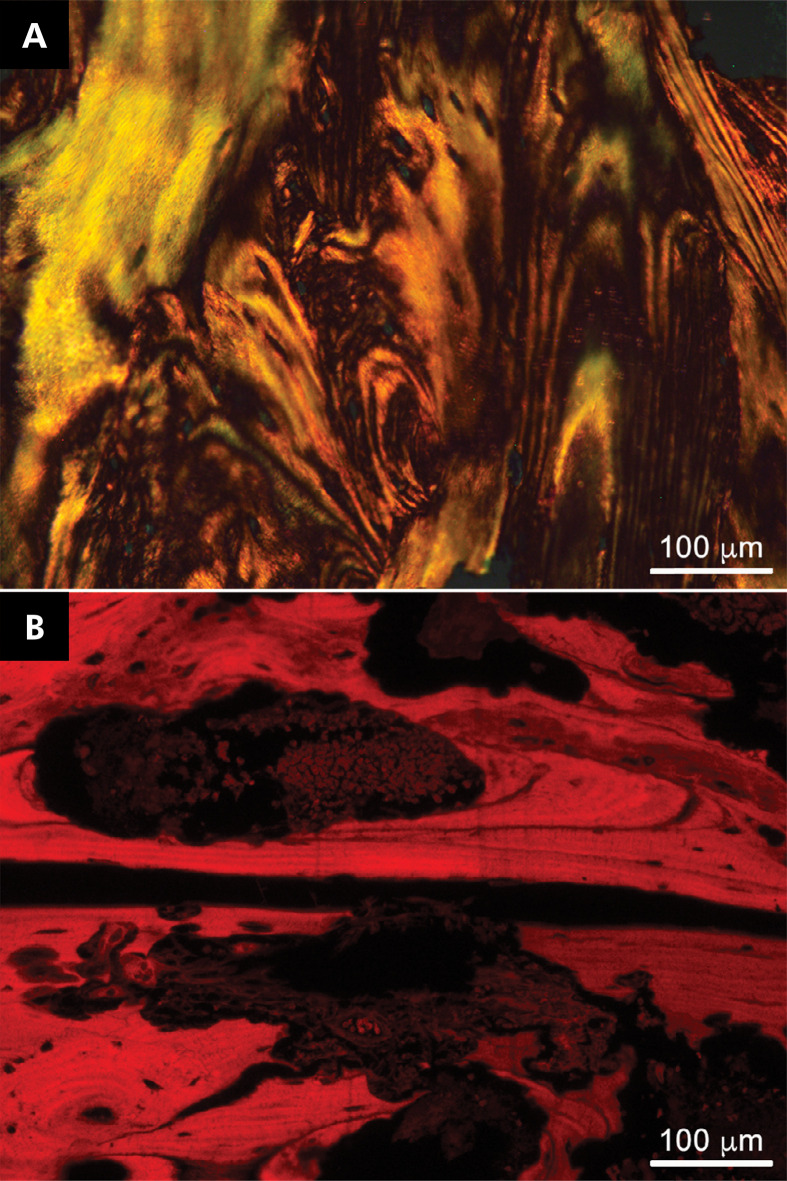
A) Polarized light microscopy of hematoxylin/eosin-stained tissues highlighting the lamellar feature [scale bar: 200 µm]. B) Medication-related osteonecrosis of the jaw showing lamellar bone and osteon-like structures with strong red fluorescence [scale bar: 200µm].

Fluorescence and polarized light microscopy analysis demonstrated lamellar bone with osteon-like structures (Figure 3).

## Discussion

This study described a series of 22 MRONJ cases. Although MRONJ is still little known, the presence of this type of osteonecrosis does not seem to be a rare finding since it has been reported over the past years.^3,26,27^ However, a description of the clinicopathological features of MRONJ is important since they can have clinical implications such as infections^12,28,29^ and pathological fractures.^2,13^ In addition, the reported suspected cases generally did not state the criteria adopted by the American Association of Oral and Maxillofacial Surgeons for diagnosing MRONJ,^5^ and some were suspected cancer cases.

In the present series, MRONJ was more frequent in the mandible of women, in agreement with previous studies.^16,19,22,30-32^ The highest frequency of MRONJ was found in patients older than 80 years, followed by those in their seventies and nineties. Thus, MRONJ affects older patients, as reported by other authors.^17,26,31^ According to Cremers and Papapoulos,^33^ the jaw bones are characterized by high turnover rates and bisphosphonates seem to be attracted to bone with this type of metabolism. However, other authors reported that bisphosphonates are deposited in bones with high mineral content, as is the case of the jaw, which contains a significant amount of hydroxyapatite.^34^

The radiographic signs of MRONJ included osteosclerosis, lysis, and bone sequestration, as previously described by the authors.^13^ Other conditions such as florid cemento-osseous dysplasia (FCOD)-related osteonecrosis exhibit similar radiograph features despite their distinct etiology and can therefore show mixed density.^25^ It is important to stress that the lack of radiographic images was a limitation of the present study. However, panoramic radiographs, as observed here, remain relatively nonspecific, although more advanced imaging may be useful.^35^

In addition to necrosis, other histopathological features of MRONJ must be highlighted, although we were unable to distinguish them based on the type of medication used since the drugs have the same pharmacological action. These features included irregular bone trabeculae with empty lacunae that were larger than in the presence of osteocytes, as well as the observation of bone resorption, inflammation, and bacterial colonization. It is important to note that MRONJ was rarely referred to clinically as a bone sequestrum, which was observed in some patients.

Resorbed bone surfaces were common findings in MRONJ, detected in more than 50% of the biopsy specimens. However, bone resorption was associated with osteomyelitis. According to Hong et al.,^31^ long-term medication use is the main risk factor for osteomyelitis.

We did not observe clearly visible osteoclasts in MRONJ, as this cell population seems to be rare.^15,17^ Bacterial colonization may be the cause of bone without osteoclasts, since bacteria can resorb bone.^36^ Nevertheless, the pharmacological action might interfere with the interaction between RANK-Fc and OPG-Fc,^37^ suppressing the activity or stimulating apoptosis of osteoclasts. This mechanism is complex, and studies on endocrine disorders and autophagy that involve the main regulator of osteoclastogenesis (nuclear factor of activated T-cells cytoplasmic 1, NFATc1)^38^ may clarify this matter.

All patients exhibited osteomyelitis of variable intensity, especially acute osteomyelitis, as was also reported by Shuster et al.^15^ Acute osteomyelitis was also observed in other case series of MRONJ.^39^ We believe that osteomyelitis is secondary to the process of necrosis, which attracts inflammatory cells that phagocytose the necrotic tissue. The host organism responds by expelling these cells, as indicated by the formation of a bone sequestrum. There appears to be an increased risk of MRONJ in the presence of root amputation, single tooth extraction, bone loss, tooth mobility, and open wounds,^11,32,40^ with the consequent development of osteomyelitis.

It is important to note that the jaws are the only skeletal bones exposed to the exogenous environment because the bone is lined only by the thin oral mucosa. This fact facilitates infarction or infection or both,^25^ which can be related to the low immunity of this process.

Previous authors have also reported bacterial colonization in patients with MRONJ.^17^ In contrast to other authors,^15,42^ we observed trapped bacteria or bacteria occupying bone marrow spaces. Although many MRONJ cases were fragmented, it is possible that the smaller trabecular thickness contributed to reaching the deeper necrotic bone tissue. We have observed that many bone trabeculae are visibly smaller in MRONJ than in other types of necrosis, such as FCOD-related osteonecrosis.^25^ These features of MRONJ specimens are compatible with other authors who reported that loss of connectivity among trabeculae negatively influences bone strength.^19^

*Actinomyces* formed single or multiple colonies that could sometimes be observed in the same specimen. Actinomycotic grains have been frequently associated with MRONJ.^15-17,41^ Some authors have reported colonization with *Actinomyces* as a secondary cause of MRONJ,^15^ while others believe it to be an opportunistic infection^16,41^ or a complication of jaw osteonecrosis.^43^ The presence of bacteria trapped inside the bone tissue does not seem to support the idea of an opportunistic infection. Although the pathogenic role of *Actinomyces* in MRONJ remains controversial,^43^ their presence could have clinical significance and help in planning the treatment as this infection has aggressive potential.^44,45^ Further studies need to be performed to clarify this matter.

An interesting finding was that bacterial colonies generally did not mix with the inflammatory process, which may indicate competition for bone resorption found in these specimens, as reported by Bastos et al., 2022.^25^ This may be a common finding in osteonecrosis. Another morphological finding was the rare presence of aspergillosis. Other authors did not describe this unusual finding. It is, therefore, always important to investigate fungi and actinomycosis in this lesion. Thus, these infections require additional therapy in MRONJ.

Basophilic reversal lines are an important feature of the bone remodeling process.^46,47^ This study found “thick and irregular” lines, as also demonstrated by Kim et al.^48^ Although some histological features such as lamellae and osteon-like structures can be found in MRONJ similar to normal bone, other methods such as fluorescence and polarized light microscopy performed in this study also revealed a more lamellar appearance of MRONJ tissues. These features are compatible with the macrolamellar pattern of the collagen fibers of bone-like structures found in MRONJ^31,46^ and are different from those seen in FCOD-related osteonecrosis.^25^

Reactive bone has been found sometimes. Some authors observed the neoformation of bone as large masse^31^, but we did not find this feature or bone remodeling in any of the patients studied. We hypothesize that the presence of viable bone represents a bone product removed during surgical resection, which was not affected by the necrotic process. In contrast to the present study, other authors observed features such as thick trabeculae and medullar spaces.^31^ However, larger diameter osteocytes were frequent.

In conclusion, this study provided important insight into MRONJ. It demonstrated a diverse histopathological spectrum, including *Actinomyces* and fungal infections, which can have important clinical implications. In addition, MRONJ was characterized by a necrotized lamellar bone structure and more frequently affected the mandible of older women.
